# The complete mitochondrial genome of *Pareuchiloglanis gongshanensis* (Siluriformes, Sisoridae, *Pareuchiloglanis*): genome characterization and phylogenetic analysis

**DOI:** 10.1080/23802359.2015.1137822

**Published:** 2016-02-05

**Authors:** Bo Li, Zhifu Tian, Ya Qin, Meng Hao, Jiabo Zhang

**Affiliations:** a College of Fisheries, Huazhong Agricultural University, Wuhan, China;; b Changjiang Water Resources Protection Institute, Wuhan, China

**Keywords:** Control region, mitochondrial genome, *Pareuchiloglanis gongshanensis*, phylogenetic relationship

## Abstract

*Pareuchiloglanis gongshanensis*, a small-sized benthic fish, distributed in southwest China. In the present study, the complete mitochondrial genome of *P. gongshanensis* was sequenced to be 16 588 bp in length, including 13 protein-coding genes, 2 ribosomal RNA genes, 22 transfer RNA genes, a control region and the origin of the light strand replication. The overall nucleotide composition was 30.70% A, 24.11% T, 29.16% C and 16.02% G, with an A + T bias of 54.82%. The gene composition and the structural arrangement of the *P. gongshanensis* complete mitochondrial DNA were identical to most of the other vertebrates. This will provide a useful tool for understanding the genetic diversity, population structure and conservation status of *P. gongshanensis* in the future.

## Introduction

Mitochondrial DNA (mtDNA) gene order was proposed to be quite conserved within vertebrates based on the gene order of the initial genome sequence (Anderson et al. 1981; Bibb et al. 1981). *Pareuchiloglanis gongshanensis* (Siluriformes: Sisoridae) is an endemic fish species which mainly distributes in the upper reaches of the Yangtze River and its tributaries in China. In recent years, the natural resource of this species has seriously declined, as a result of overharvesting, water contamination and especially dam construction. In the long run, a good understanding of the genetic diversity and population structure of *P. gongshanensis* is required in order to establish adequate management plans for the conservation of this species. To address these topics, we determined the complete mitochondrial genome sequence of *P. gongshanensis* for the first time.

Specimens of *P. gongshanensis* were collected from Nujiang River, Yunnan Province in March 2015 and preserved in 95% ethanol until total genomic DNA was isolated from the caudal fin by proteinase K digestion followed by the standard phenol/chloroform method (Sambrook and Russell, 2001) and visualized on 1.5% agarose gel. Twenty sets of primers were designed for PCR amplification on the basis of aligned mitogenome sequences of *Pareuchiloglanis sinensis* with (Accession NC_024434.1). In order to avoid errors of assembly, the complete mtDNA genome was aligned and checked with four reported mtDNA genome sequences of Sisoridae species *Creteuchiloglanis kamengensis* (Accession NC_021599.1); *Oreoglanis macropterus* (Accession NC_021607.1); *Pseudexostoma yunnanensis* (Accession NC_021604.1) and *Pareuchiloglanis gracilicaudata* (Accession NC_021603.1). The assembled sequence was analyzed using the software MitoAnnotator (Iwasaki et al. 2013) and nucleotide composition was calculated by MEGA6 (Tamura et al. 2013).

The complete mtDNA sequence of *P. gongshanensis* reported here has been deposited in GenBank under the accession number KU160626. The mitochondrial genome of *P. gongshanensis* is a circular molecule of 16 588 nucleotides, which is similar to other vertebrates, including 13 protein-coding genes, 2 ribosomal RNA genes, 22 transfer RNA (tRNA) genes and a non-coding control region (D-loop). The overall nucleotide composition is 30.70%, 24.11%, 29.16% and 16.02% for A, T, C and G, with an A + T content of 54.82%, respectively. Except for a single protein-coding gene (ND6) and eight tRNA genes (tRNA*^Gln^*, tRNA*^Ala^*, tRNA*^Asn^*, tRNA*^Cys^*, tRNA*^Tyr^*, tRNA*^Ser^* [UCN], tRNA*^Glu^* and tRNA*^Pro^*) encoded on the L-strand. All the other genes were encoded on the H-strand. The first non-coding region is 894bp between tRNA*^Pro^* and tRNA*^Phe^*, and the second one is the origin of light-strand replication, which extends up to 31 bp. It is located in a cluster of 5′-tRNA genes (the WANCY region) between tRNA*^Asn^* and tRNA*^Cys^* genes.

Furthermore, the termination codon varies with TAA, TA, T or TAG. Virtually, all of the 13 protein-coding genes show the regular initiation codon ATG with the sole exception of COI and ND3 which started with GTG. Six protein-coding genes terminated with the complete stop codon TAA (ND1, COI, ATPase8, ND4L and ND5) or TAG (ND6), while the rest ended with incomplete stop codon T (ND2, COII, COIII, ND3, ND4 and Cytb) or TA (ATPase 6) which is quite typical among mtDNA genes in other fishes (Zhou et al. 2012; Wang et al. 2013).

**Figure 1.**
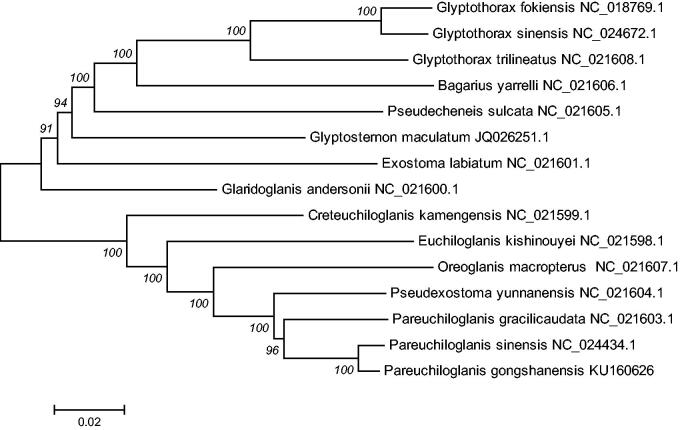
The consensus phylogenetic relationship of the *Pareuchiloglanis gongshanensis* with other Sisoridae species. *Glyptothorax fokiensis*, *Glyptothorax sinensis*, *Glyptothorax trilineatus* and *Bagarius yarrelli* were used as out-group. The numbers along the branches are Bayesian posterior probability and bootstrap values for neighbor-joining method, estimated for concatenated mitochondrial protein sequences.

In addition, the mtDNA sequences of 14 species of fishes were downloaded from GenBank, *Bagarius yarrelli* (Accession NC_021606.1), *Glyptothorax fokiensis* (Accession NC_018769.1), *Glyptothorax sinensis* (Accession NC_024672.1), *Glyptothorax trilineatus* (Accession NC_021608.1) were used as an out-group for phylogenetic analysis. Phylogenetic analyses were performed using the neighbor-joining method in MEGA 6.0 (Kumar et al. 2008). The tree topologies based on complete mtDNA sequences in this study were identical and were statistically supported by high bootstrap and posterior probability values (Figure 1). The mitogenome data provided strong support that *P. Gongshanensis* was clustered together and form a sister group with *P. sinensis* (Accession NC_024434.1), *P. gracilicaudata* (Accession NC_021603.1), *P. yunnanensis* (KP872696.1), *O. macropterus* (Accession NC_021607.1), *Euchiloglanis kishinouyei* (Accession NC_021598.1), *C. kamengensis* (Accession NC_021599.1). The phylogenetic analyses yielded convincing evidence that the *P. gongshanensis* is located at the bottom position in *Pareuchiloglanis* species, and that the tandem repeats might be a feature of the ancestral teleost lineage.
